# Comprehensive Proteomics Analysis Identifies CD38-Mediated NAD^+^ Decline Orchestrating Renal Fibrosis in Pediatric Patients With Obstructive Nephropathy

**DOI:** 10.1016/j.mcpro.2023.100510

**Published:** 2023-02-17

**Authors:** Yuandong Tao, Jifeng Wang, Xuexue Lyu, Na Li, Dong Lai, Yuanyuan Liu, Xingyue Zhang, Pin Li, Shouqing Cao, Xiaoguang Zhou, Yang Zhao, Lifei Ma, Tian Tao, Zhichun Feng, Xiubin Li, Fuquan Yang, Huixia Zhou

**Affiliations:** 1 Department of Pediatric Urology, Senior Department of Pediatrics, The Seventh Medical Center of Chinese PLA General Hospital, Beijing, China; 2 National Engineering Laboratory for Birth Defects Prevention and Control of Key Technology, Beijing, China; 3 Laboratory of Proteomics & Key Laboratory of Protein and Peptide Pharmaceuticals Institute of Biophysics, Chinese Academy of Sciences, Beijing, China; 4 University of Chinese Academy of Sciences, Beijing, China; 5 Medical School of Chinese PLA, Beijing, China; 6 Department of Urology, The Third Medical Center of Chinese PLA General Hospital, Beijing, China; 7 Department of Dermatology, The Seventh Medical Center of Chinese PLA General Hospital, Beijing, China; 8 College of Graduate, Hebei North University, Zhangjiakou, China

**Keywords:** obstructive nephropathy, renal fibrosis, kidney proteomics, NAD^+^, CD38, CKD, chronic kidney disease, DIA, data-independent acquisition, FAO, fatty acid oxidation, MS, mass spectrometry, NAM, nicotinamide, PARP, poly (ADP-ribose) polymerase, ROC, receiver operating characteristic curves, SIRT, sirtuin, TGF, transforming growth factor, UPJO, ureteropelvic junction obstruction, UUO, unilateral ureteral obstruction

## Abstract

Obstructive nephropathy is one of the leading causes of kidney injury and renal fibrosis in pediatric patients. Although considerable advances have been made in understanding the pathophysiology of obstructive nephropathy, most of them were based on animal experiments and a comprehensive understanding of obstructive nephropathy in pediatric patients at the molecular level remains limited. Here, we performed a comparative proteomics analysis of obstructed kidneys from pediatric patients with ureteropelvic junction obstruction and healthy kidney tissues. Intriguingly, the proteomics revealed extensive metabolic reprogramming in kidneys from individuals with ureteropelvic junction obstruction. Moreover, we uncovered the dysregulation of NAD^+^ metabolism and NAD^+^-related metabolic pathways, including mitochondrial dysfunction, the Krebs cycle, and tryptophan metabolism, which led to decreased NAD^+^ levels in obstructed kidneys. Importantly, the major NADase CD38 was strongly induced in human and experimental obstructive nephropathy. Genetic deletion or pharmacological inhibition of CD38 as well as NAD^+^ supplementation significantly recovered NAD^+^ levels in obstructed kidneys and reduced obstruction-induced renal fibrosis, partially through the mechanisms of blunting the recruitment of immune cells and NF-κB signaling. Thus, our work not only provides an enriched resource for future investigations of obstructive nephropathy but also establishes CD38-mediated NAD^+^ decline as a potential therapeutic target for obstruction-induced renal fibrosis.

## Highlights

• Proteomics uncovered a profibrotic and inflammatory phenotype in obstructed kidneys.
• Obstruction induced a global metabolic dysregulation and an aberrant NAD^+^ metabolism.
• CD38 was strongly induced in human and experimental obstructive nephropathy.
• CD38 deletion or inhibition mitigated obstruction-induced renal fibrosis.

## Graphical Abstract

**Figure:**
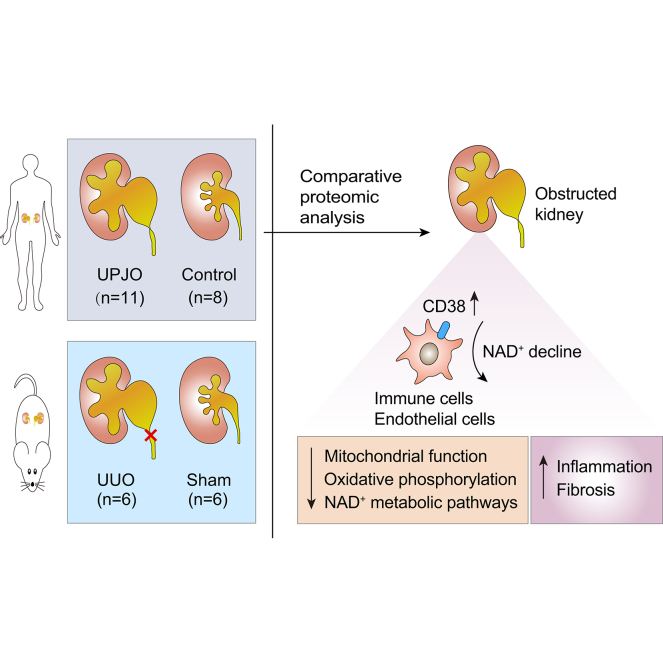

## In Brief

Obstructive nephropathy is a leading cause of kidney injury in infants and children. In this work, we performed comparative proteomics of control and obstructed kidneys from human and experimental obstructive nephropathy and uncovered the aberrant NAD^+^ metabolism, which was partially induced by CD38 upregulation. Deletion or inhibition of CD38 reduced obstruction-associated renal fibrosis and inflammation. These findings emphasized the therapeutic potential of CD38 and NAD^+^ metabolism in obstructive nephropathy.

Obstructive nephropathy is a leading cause of kidney injury in infants and children, which is frequently induced by hydronephrosis in pediatric patients with ureteropelvic junction obstruction (UPJO) (1, 2, 3). Prolonged obstructive hydronephrosis leads to chronic kidney disease (CKD) and renal fibrosis, characterized by an excessive extracellular matrix accumulation and the progressive loss of kidney function (4). To date, effective antifibrotic therapies are still lacking. Therefore, it is critical to expanding our understanding of obstructive nephropathy to facilitate the development of new treatments.

The current understanding of the pathogenesis of obstruction-induced kidney injury is mostly derived from animal experiments, for instance, the unilateral ureteral obstruction (UUO) model (5). It is believed that obstructive hydronephrosis is associated with a higher intrapelvic and intratubular hydrostatic pressure, which stimulates apoptosis and necrosis of tubular cells and the resultant release of damage-associated molecular patterns (6). These lead to the infiltration of immune cells, including macrophages and neutrophils, and the synthesis of proinflammatory cytokines and profibrotic factors, such as interleukin-1 beta (IL-1β), tumor necrosis factor-alpha (TNF-α), transforming growth factor-beta (TGF-β), and platelet-derived growth factor (PDGF). The profibrotic microenvironment promotes the activation of myofibroblasts, the major source of extracellular matrix, thus driving the development of renal fibrosis (7). Although these findings have provided important insights into obstructive nephropathy, the etiology of hydronephrosis-induced kidney injury in pediatric patients is largely unknown, and only large-scale, unbiased discovery experiments can enable the assessment of multiple biological processes, networks, and regulators simultaneously.

Nicotinamide adenine dinucleotide (NAD^+^) is an essential cofactor for various metabolic processes, such as glycolysis, the Krebs cycle, and fatty acid oxidation (FAO) (8). The cellular NAD^+^ can be synthesized from the essential amino acid tryptophan *via* the *de novo* pathway as well as from dietary nicotinic acid *via* the Preiss–Handler pathway. Alternatively, NAD^+^ can be produced from different forms of vitamin B3 *via* the salvage pathway (9, 10). The NAD^+^ consumption is catalyzed by three classes of enzymes: sirtuins (SIRTs), poly (ADP-ribose) polymerases (PARPs), and cyclic ADPribose synthetases (CD38) (11). Interestingly, recent studies indicate that NAD^+^ is implicated in the development of kidney diseases (8). A rapid decrease in NAD^+^ levels is observed in acute kidney injury, which is partially driven by impaired do novo NAD^+^ biosynthesis (12, 13). Recovery of NAD^+^ levels *via* supplementation with NAD^+^ precursors, such as nicotinamide mononucleotide or nicotinamide (NAM), protects against kidney damage in acute kidney injury models (12, 14, 15). Moreover, several studies in animals and clinical observations suggest a linkage between NAD^+^ metabolism and the development of CKDs (8). However, few data are available on the details of NAD^+^ metabolism and its roles and underlying mechanisms in CKDs, including obstruction-induced renal fibrosis (10).

The multifunctional NADase CD38 is a kind of ADP-ribosyl cyclase that degrades NAD^+^ and modulates cellular NAD^+^ homeostasis (16). It is most highly expressed in immune cells, such as macrophages, T cells, B cells, and monocytes (17). CD38 has a central role in age-related NAD^+^ decline in mammals, depending on its ectoenzyme or endoenzyme activity (18). Of note, recent studies reported that CD38 deficiency induces autoimmune characteristics and kidney damage in 16-month-old mice (19) and CD38 inhibition by apigenin reduces renal injury in diabetic rats through the restoration of the NAD^+^/NADH ratio (20). These findings suggest tantalizing links of CD38 and NAD^+^ to kidney diseases. Nonetheless, the roles and underlying mechanisms of CD38 in obstructive nephropathy are completely unknown.

In the present study, we analyzed the global proteome of obstructed kidneys from patients and UUO mice. We uncovered the dysregulation of NAD^+^ metabolism in obstructive nephropathy and found that NADase CD38 promoted obstruction-induced renal fibrosis and kidney inflammation.

## Experimental Procedures

### Patient Samples

The human kidney samples used in this study were obtained from The Seventh Medical Center of Chinese PLA General Hospital, with the approval of the Research Ethics Committee of the hospital. Written informed consent was provided by legal parents. Eleven obstructed kidneys were from pediatric patients with late-presented hydronephrosis induced by UPJO during nephrectomy. Eight control kidneys were sampled from the tumor-free kidney cortex 5 cm away from the tumor region of patients with nephroblastoma during radical nephrectomy. Before nephrectomy in both UPJO and tumor cases, the kidney vessels were clamped within 15 min. The operations were performed by a single skilled surgeon. Surgically resected kidney tissues were frozen in liquid nitrogen for storage before use. The clinical information of patients was shown in Table 1.

**Table 1:** Clinical information of patients

Characteristics	Obstructed kidneys (n = 11)	Control kidneys (n = 8)	*p* value
Age (months) (mean) [s.d.]	25.85 (22.53)	48.23 (44.26)	0.220
Gender			0.040
Male	3	6	
Female	8	2	
Degree of hydronephrosis			0
0	0	8	
1	0	0	
2	0	0	
3	0	0	
4	11	0	
Diagnosis	Hydronephrosis	Nephroblastoma	
Operation side			0.599
Left	4	2	
Right	7	6	
BUN (mM) (mean) [s.d.]	4.58 (1.86)	4.00 (1.39)	0.473
Cre (mM) (mean) [s.d.]	34.50 (6.64)	38.78 (7.62)	0.210

Abbreviations: BUN, serum urea nitrogen; Cre, creatinine.

### Mice and UUO Model

C57BL/6 wildtype mice were purchased from Charles River in Beijing (Vital River). *Cd38*^−/−^ mice on a C57BL/6 background were kindly provided by Dr Xingyue Zhang (The Seventh Medical Center of Chinese PLA General Hospital). All animal experimental procedures were approved by the Institutional Animal Care and Utilization Committees of the Chinese PLA General Hospital. All mice were maintained in a specific pathogen-free condition. For the UUO model, male mice aged between 7 and 8 weeks were used. Generally, a median abdominal incision was performed after anesthetization and the left ureter was double ligated. The sham group underwent the same procedure except for the ureteral ligation. Mice were sacrificed 7 days (unless specified otherwise) after surgery and kidneys were then harvested.

For the inhibition of CD38, compound 78c (S8960, Selleck) was administrated *via* oral gavage for six consecutive days (200 μg/mouse/day). NAD^+^ (N7004, Sigma) was supplemented by peritoneal injection (100 μg/mouse/day).

### Sample Preparation

The kidney tissues were disrupted by using a Bertin homogenizer on ice in lysis buffer (8 M urea/0.1 M Tris-HCl, pH 8.0) containing 1× Protease Inhibitor Cocktail (Roche). After centrifugation, the extracted proteins were reduced with 10 mM DTT for 2 h at room temperature followed by alkylation with 20 mM iodoacetamide for 30 min in the dark. Samples were then digested with trypsin (1:50) at 37 °C overnight. The digestion was desalted on an OASIS HLB column, and peptides eluted with 60% acetonitrile were lyophilized *via* vacuum centrifugation and dissolved in 0.1% formic acid before mass spectrometry (MS) data acquisition.

### Data-Independent Acquisition Mass Spectrometry Data Acquisition

All nano-liquid chromatography tandem mass spectrometry experiments were performed on Orbitrap Eclipse (Thermo Scientific) equipped with an Easy n-LC 1200 HPLC system (Thermo Scientific). The peptides were loaded onto a 100 μm id × 2 cm fused silica trap column packed in-house with reversed-phase silica (Reprosil-Pur C18 AQ, 5 μm, Dr. Maisch GmbH) and then separated on a 75 μm id × 25 cm C18 column packed with reversed-phase silica (Reprosil-Pur C18 AQ, 1.9 μm, Dr. Maisch GmbH). The peptides bounded on the column were eluted with a 103-min linear gradient. Solvent A consisted of 0.1% formic acid in water solution, and solvent B consisted of 80% acetonitrile and 0.1% formic acid. The segmented gradient was 4 to 11% B, 4 min; 11 to 21% B, 28 min; 21 to 30% B, 29 min; 30 to 42% B, 27 min; 42 to 99% B, 5 min; 99% B, 10 min at a flow rate of 300 nl/min.

The MS analysis was performed with Orbitrap Eclipse mass spectrometer (Thermo Scientific). With the data-independent acquisition mode, the MS data were acquired at a high resolution 120,000 (*m/z* 200) across the mass range of 400 to 1210 *m/z*. The target value was 4.00E+05 with a maximum injection time of 50 ms. One full scan was followed by 40 windows with an isolation width of 16 *m/z* for fragmentation in the Ion Routing Multipole with HCD normalized collision energy of 30%. Tandem mass spectrometry spectra were acquired at resolution of 30,000 at *m/z* 200 across the mass range of 200 to 2000 *m/z*. The target value was 4.00E+05 with a maximum injection time of 50 ms. For the nanoelectrospray ion source setting, the spray voltage was 2.0 kV; no sheath gas flow; the heated capillary temperature was 320 °C.

### Data-Independent Acquisition Data Analysis

The DIA raw data from Orbitrap Eclipse were analyzed using Spectronaut version 14 (Biognosys) with the “DirectDIA” mode for protein identification and quantification. The UniProt human or mouse proteome database (download date 2021-07-05) was used for searching the data from kidney samples, and the total number of database entries searched for human and mouse was 20,371 and 55,341, respectively. The most important searching parameters were set as the default settings: trypsin was selected as enzyme and two missed cleavages were allowed for searching; the mass tolerance of MS1 and MS2 was set as correction factor 1; cysteine carbamidomethylation was specified as fixed modification; the methionine oxidation and acetylation of protein N-term were chosen as variable modifications. False discovery rate <1% was set for peptide spectrum matches, peptides, and proteins identification. The data were filtered by Qvalue, and the “Global Normalization” was set as “Median” with enabled cross run normalization. Statistical analyses of proteins were performed in Spectronaut. Two-sample *t* test was used for the calculation of *p* value, and the resulting *p* value was adjusted using the Benjamini–Hochberg method. Proteins with *p* value <0.05, *q* value <0.05, and fold change >2 or <0.5 were considered as up- or downregulated differentially expressed proteins.

### Western Blots

Kidney protein extracts were prepared according to standard protocols (21). Cell lysates were separated by 10% SDS-PAGE and transferred to polyvinylidene difluoride membranes (Millipore). The following antibodies were used (all from Cell Signaling Technology unless specified otherwise): monoclonal mouse anti-mouse/human CD38 (sc-374650, Santa Cruz), monoclonal mouse anti-mouse GAPDH (5174T), monoclonal rabbit anti-mouse/human SMAD2 (5339T) and phospho-SMAD2 (18338T), monoclonal rabbit anti-mouse/human NF-κB p65 (8242T) and phospho-NF-κB p65 (3033T).

### Immunohistochemistry

Human and mouse kidneys were fixed in 4% paraformaldehyde and embedded with paraffin. The kidney sections were stained with hematoxylin and eosin, Masson's trichrome, and rabbit anti-mouse αSMA antibody (19245, Cell Signaling Technology). Images of kidney slides were obtained on a Nano Zoomer Slide Scanner (Hamamatsu Photonics). The percentages of collagen-positive areas and αSMA-positive areas were quantified by ImageJ software.

### Real-Time Quantitative PCR

Mouse kidneys were homogenized and total RNA was extracted using Trizol according to the manufacturer’s protocol (Thermo Fisher). Complementary DNA was generated using a Reverse Transcription kit (Takara). Real-time quantitative PCR was performed using the iCycler iQ5 Real-Time PCR detection system (Bio-Rad). The expression of the target gene was normalized to the expression of the housekeeping gene, *Gapdh*. Relative gene expression was calculated using the standard 2^−ΔΔCt^ method. The primers used were shown in Table 2.

**Table 2:** Primers for real-time quantitative PCR

Genes	Forward	Reverse
m*Gapdh*	ATCTTCTTGTGCAGTGCCAGC	GTTGATGGCAACAATCTCCAC
m*Tgfb1*	CGCAACAACGCCATCTATGA	ACTGCTTCCCGAATGTCTGA
m*Il1b*	TGTAATGAAAGACGGCACACC	TCTTCTTTGGGTATTGCTTGG
m*Kim1*	CTATGTTGGCATCTGCATCG	AAGGCAACCACGCTTAGAGA
m*Ngal*	GATGAACTGAAGGAGCGATTC	TCGGTGGGAACAGAGAAAAC

### Kidney Leukocyte Isolation and Flow Cytometry Analysis

Mice under indicated conditions were anesthetized, and kidneys were harvested and cut into pieces. Then kidney tissues were digested in a buffer (HBSS supplemented with 0.05% collagenase I and 2 mM CaCl_2_) under 37 °C for 25 min. After digestion, the kidney tissues were filtered through a 70-μm nylon mesh. The cell suspension was centrifuged at 500*g* for 5 min, and the resulting cell suspension was treated with Fcγ receptor blocker (101320, BioLegend) for 10 min followed by incubating with the following fluorescent antibodies (all from BioLegend): CD45 BV421 (103134), CD11b FITC (101206), Ly6G APC/Cyanine7 (127624), Ly6C PE (128008), F4/80 APC (123116), 7AAD (420404), and CD38 PE/Cyanine7 (102717). Flow cytometry was performed on a FACSCanto II (BD Biosciences), and data were analyzed by FlowJo software 10.4.

### Renal NAD^+^ Detection

NAD^+^ levels in mouse kidney tissues were measured using a NAD/NADH quantification kit (MAK037, Sigma) according to the manufacturer’s protocol.

### Experimental Design and Statistical Rationale

The study aimed to obtain a comprehensive understanding of human obstructive nephropathy at the proteome level. The experimental design is shown in Graphical Abstract. Eleven human obstructed kidneys and eight control kidneys were used to perform the proteomic study. Mouse kidney proteomics was performed with six sham and six UUO kidneys. The number of replicates and biological and statistical methods used for analysis were described in the figure legends. All tissues were lysed and digested in parallel. Data were presented as mean ± SEM. Statistical analyses were performed with GraphPad Prism version 6.0c or R version 4.1. Two-tailed Student’s *t* test was used for comparisons between two groups. Spearman's rank correlation was used to evaluate relationships between two variables. A *p* value less than 0.05 was considered significant. The receiver operating characteristic (ROC) curve analysis was performed in the pROC package.

## Results

### Proteomic Profiling of Human Obstructed Kidneys

**Fig. 1:**
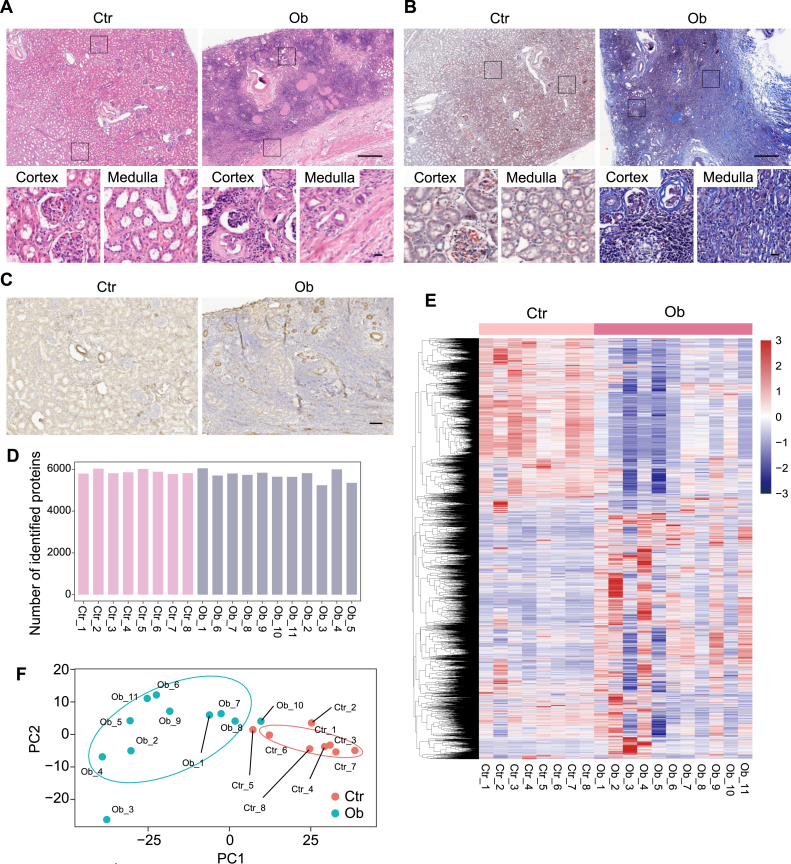
**Proteomic profiling of human obstructed kidneys.***A*–*C*, representative images of hematoxylin & eosin (H&E) staining (*A*), Masson's trichrome staining (*B*), and αSMA immunohistochemistry staining (*C*) of kidney sections from control and patients with obstructive nephropathy. The scale bars represent: (*A* and *B*) upper panels 400 μm, lower panels 20 μm, (*C*) 100 μm. *D*, protein numbers identified by data-independent acquisition mass spectrometry in kidney samples obtained from control and patients with obstructive nephropathy. *E*, heatmap indicating all proteins identified by data-independent acquisition mass spectrometry in kidneys from control and patients with obstructive nephropathy. *F*, principle component analysis of control kidney proteomes (n = 8) and obstructed kidney proteomes (n = 11).

The study collected 11 obstructed kidneys (Ob) from pediatric patients with hydronephrosis and eight control kidneys (Ctr) of peritumor tissues from pediatric patients with nephroblastoma (Table 1). Histological examination revealed tubule atrophy, urinary cast, and leukocyte infiltration in obstructed kidneys (Fig. 1*A*). Moreover, the obstructed kidneys showed excessive collagen deposition (Fig. 1*B*) and an increased number of αSMA-positive cells compared with controls (Fig. 1*C*). These results indicate that sustained ureteral obstruction led to kidney injury and renal fibrosis.

To obtain an extensive molecular understanding of human obstructive nephropathy, a DIA-MS approach was used to perform the proteomic study. Proteomics measurement of all human kidney samples resulted in a total of 6258 proteins with an average of 5780 proteins per sample (Fig. 1, *D* and *E*). Principle component analysis demonstrated a clear boundary between the two proteomes (Fig. 1*F*), indicating an abnormal proteomic landscape in the kidneys from patients with obstructive nephropathy.

### Proteomic Landscape of Human Obstructed Kidneys

**Fig. 2:**
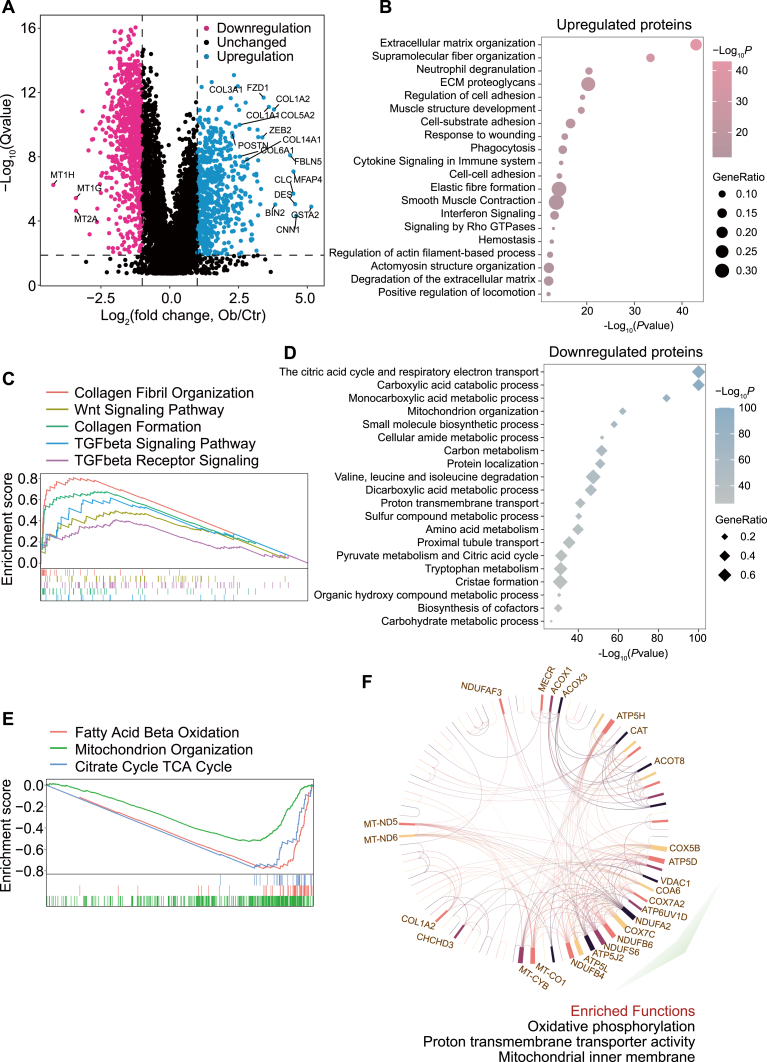
**Proteomic landscape of human obstructed kidneys.***A*, volcano plot generated by differential analysis of the proteomic profiles of obstructed kidneys *versus* control kidneys. Significantly upregulated proteins were shown as blue dots and downregulated proteins were shown as pink dots (*p* value <0.05, *q* value <0.05, and fold change >2 or <0.5). *B*, top 20 most significant terms enriched by upregulated proteins in obstructed kidneys. *C*, gene set enrichment analysis showing pathways significantly upregulated in kidneys from patients with obstructive nephropathy. *D*, top 20 most significant terms enriched by downregulated proteins in obstructed kidneys. *E*, gene set enrichment analysis showing pathways dramatically downregulated in kidneys from patients with obstructive nephropathy. *F*, receiver operating characteristic curve analysis was performed and proteins with area under ROC curve = 100% were used for protein–protein interaction analysis and Gene Ontology enrichment analysis.

Next, differentially expressed proteins between control and obstructed kidneys were identified. Compared with the control, 614 proteins were upregulated and 855 proteins were downregulated (*p* value <0.05, *q* value <0.05, and fold change >2 or <0.5) in human obstructive nephropathy (Fig. 2*A*). Functional enrichment analyses were performed by using Metascape (22) to identify the altered biological processes and signaling pathways in human obstructed kidneys. The upregulated proteins were significantly enriched in terms of collagen formation and immune response (Fig. 2*B*), which was consistent with the histological results (Fig. 1, *A*–*C*). Several collagens were dramatically increased in kidneys from patients with obstructive nephropathy, such as COL1A1/2, COL6A1, COL5A2, and COL3A1 (Fig. 2*A* and supplemental Fig. S1*A*). POSTN (23), a previously demonstrated signature protein for renal fibrosis, exhibited a 5-fold increase in obstructed kidneys (Fig. 2*A*). In line with previous studies demonstrating that inflammation is a critical contributor to renal fibrosis (24, 25), our study showed significant enrichment of neutrophil response, phagocytosis, cytokine signaling, and interferon signaling in the upregulated proteins (Fig. 2*B* and supplemental Fig. S1, *B* and *C*). Furthermore, gene set enrichment analysis revealed substantial upregulation of collagen fibril organization and collagen formation, as well as a significant increase in TGF-β signaling and Wnt signaling in obstructed kidneys (Fig. 2*C*), which are strongly associated with the development of renal fibrosis (26). Supporting this, kidneys from individuals with obstructive nephropathy showed increased phosphorylation of SMAD2 (supplemental Fig. S1*D*). Taken together, these results indicate that prolonged ureteral obstruction induces a fibrotic and inflammatory phenotype in human kidneys.

However, the downregulated proteins in obstructed kidneys were significantly enriched in the biological processes of proximal tubule transport and proton transmembrane transport, suggesting an impairment of renal function induced by ureteral obstruction (Fig. 2*D*). Furthermore, proteomics revealed extensive metabolic reprogramming events in obstructed kidneys. Biological processes including the Krebs cycle, mitochondrion organization, carbohydrate metabolic process, and tryptophan metabolism were remarkably enriched in the downregulated proteins (Fig. 2*D*). Meanwhile, gene set enrichment analysis revealed dramatic downregulation of mitochondrion organization and the Krebs cycle in kidneys from patients with obstructive nephropathy (Fig. 2*E* and supplemental Fig. S2). We also observed a dramatic downregulation of FAO in obstructed kidneys (Fig. 2*E*), validating the previous study showing that defective FAO in renal tubular epithelial cells has a key role in kidney fibrosis development (27). Then, we performed ROC curves analysis on the differentially expressed proteins to identify the most discriminant proteins (area under the ROC curve = 100%) between control and obstructed kidneys. Interestingly, proteins with area under the ROC curve = 100% were found to be mainly enriched in oxidative phosphorylation and mitochondrial function (Fig. 2*F*), suggesting that mitochondrial dysfunction might be a hallmark of obstruction-induced kidney injury. These results were consistent with the previous findings that mitochondrial abnormalities are critical features in the pathogenesis of several kidney diseases (28, 29). Collectively, these data indicate that ureteral obstruction leads to extensive metabolic alterations and renal dysfunction in human kidneys.

To reproduce the phenotype in animal experiments, we took advantage of the UUO mouse model, a well-established model to study obstructive nephropathy and renal fibrosis (30). The proteomics revealed tremendous changes between sham and UUO kidneys (supplemental Fig. S3, *A* and *B*). Similarly to human proteomics, substantial enrichment of extracellular matrix organization and inflammation were identified in the upregulated proteins (*p* value <0.05, *q* value <0.05, and UUO *versus* sham fold change >2 or <0.5) (supplemental Fig. S3*C*). Moreover, we detected a global dysregulation of cellular metabolism in mouse fibrotic kidneys as well, such as mitochondrial organization, the Krebs cycle, and fatty acid metabolism (supplemental Fig. S3*D*). Therefore, the UUO mouse model replicated the phenotype of human obstructive nephropathy proteomically.

### Aberrant NAD^+^ Metabolism in Human and Mouse Obstructed Kidneys

**Fig. 3:**
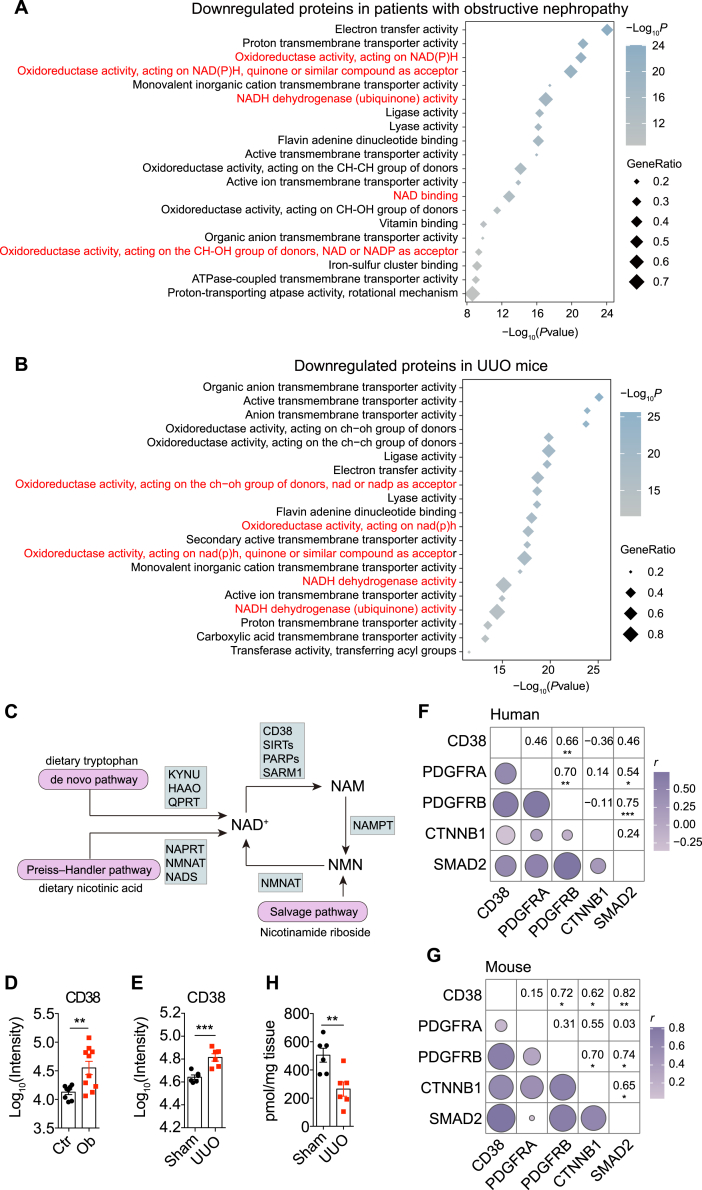
**Aberrant NAD**^**+**^**metabolism in human and mouse obstructed kidneys.***A* and *B*, top 20 most significant terms enriched by downregulated proteins in obstructed kidneys from patients with obstructive nephropathy (*A*) and UUO mice (*B*) by quarrying STRING database. *C*, schematic of cellular NAD^+^ metabolism showing key enzymes catalyzing NAD^+^ production and consumption. *D*, protein levels of CD38 in kidneys from control and patients with obstructive nephropathy. *E*, protein levels of CD38 in mouse control and obstructed kidneys. *F* and *G*, Spearman's rank correlation between the expression of CD38 and fibrosis markers in human (*F*) and mouse (*G*) kidney proteomics. *H*, tissue levels of NAD^+^ in kidneys from mice subjected to sham or UUO for 7 days. Representative data of three independent experiments. The results represent mean ± SEM. ∗*p* < 0.01, ∗∗*p* < 0.01, ∗∗∗*p* < 0.001. *p* values were calculated by two-tailed Student’s *t* test (*D*, *E* and *H*) and Spearman's rank correlation analysis (*F* and *G*). UUO, unilateral ureteral obstruction.

Since the above-identified metabolic dysregulations, including the Krebs cycle, mitochondrial dysfunction, oxidative phosphorylation, and tryptophan metabolism (Fig. 2*E*), are always associated with NAD^+^ levels (10), we asked whether NAD^+^ metabolism altered during obstructive nephropathy. Strikingly, by quarrying STRING database we found that enriched Gene Ontology molecular functions in the downregulated proteins were frequently related to NAD^+^ metabolism, in both human and experimental obstructive nephropathy (Fig. 3, *A* and *B*).

To determine if NAD^+^ homeostasis altered during the development of obstructive nephropathy, we then focused on the analysis of NAD^+^ metabolic enzymes (Fig. 3*C*). Several key enzymes catalyzing NAD^+^ *de novo* biosynthesis were dramatically decreased in human and mouse obstructed kidneys, such as HAAO and QPRT (supplemental Fig. S4). NAPRT and NMNAT3, mediating NAD^+^ synthesis *via* the salvage pathway, were significantly downregulated in patients as well as in UUO mice (supplemental Fig. S4). As for the NAD^+^ consumer, SIRT3 and SIRT5 were downregulated in both human and mouse obstructed kidneys, while SIRT2 slightly decreased only in patients (supplemental Fig. S4). PARPs showed different expression patterns between patients and mice. The human PARP4 was downregulated, whereas mouse PARP4 was upregulated in obstructed kidneys (supplemental Fig. S4). The proteomics identified unchanged expression of human PARP9, PARP10, and PARP14; however, mouse PARP9 and PARP10 were significantly increased in UUO kidneys (supplemental Fig. S4). Notably, CD38, the major NADase in mammals, was strongly induced in human and experimental obstructive nephropathy (Fig. 3, *D* and *E*). Moreover, CD38 was significantly correlated with PDGFRβ in human and mouse obstructed kidneys (*r* = 0.66, *p* < 0.01; *r* = 0.72, *p* < 0.05, respectively) (Fig. 3, *F* and *G*). PDGFRβ is a potent marker of myofibroblasts, which are the primary source of extracellular matrix in renal fibrosis (31). CD38 was also positively correlated with CTNNB1 and SMAD2 in UUO mice, the key downstream component of Wnt and TGF-β signaling, respectively (32) (Fig. 3*G*). Therefore, the proteomic study reveals an aberrant NAD^+^ metabolism and a fibrosis-associated upregulation of CD38 in obstructive nephropathy. To determine whether dysregulated NAD^+^ metabolism resulted in altered NAD^+^ content in kidneys, we then assessed renal NAD^+^ levels in UUO mice. As expected, a significant decrease in NAD^+^ levels was observed in UUO kidneys (Fig. 3*H*), demonstrating that dysregulated NAD^+^ metabolism diminished renal NAD^+^ levels.

### The Elevated CD38 Contributed to NAD^+^ Decline in Obstructed Kidneys

**Fig. 4:**
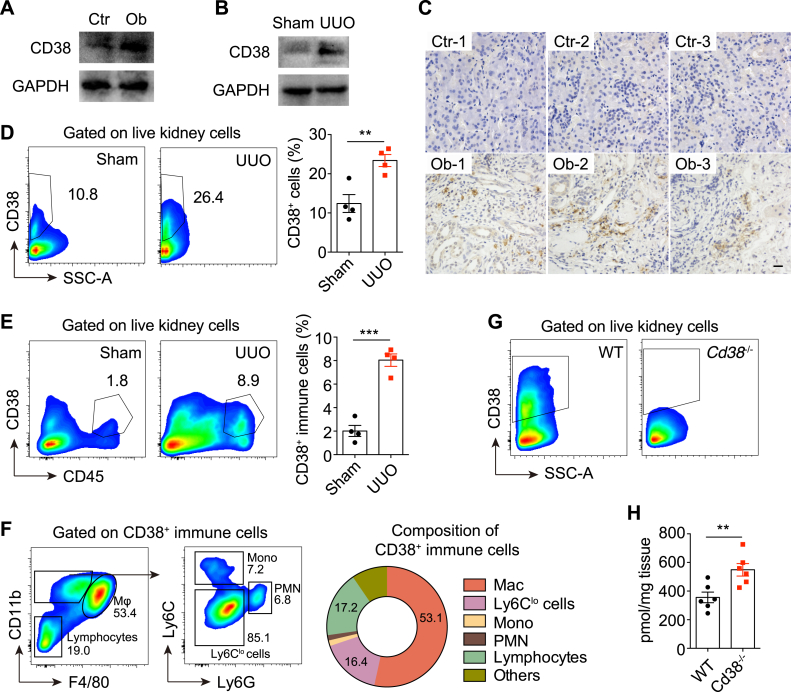
**The elevated CD38 contributed to NAD**^**+**^**decline in obstructed kidneys.***A* and *B*, CD38 protein levels in human (*A*) and mouse (*B*) control and obstructed kidneys were determined by Western blots. Represented results of three separate experiments. *C*, representative images of kidney sections from control and patients with obstructive nephropathy were stained for CD38. The scale bar represents 20 μm. *D*–*F*, flow cytometry analysis of kidneys from mice subjected to sham or UUO operation for 7 days (n = 4). The experiment was repeated three times. Quantification of CD38^+^ cells (*D*), CD38^+^ immune cells (*E*), and composition of CD38^+^ immune cells (*F*) were shown. *G*, flow cytometry analysis indicating CD38^+^ cells in obstructed kidneys from wildtype (WT) or *Cd38*^−/−^ mice subjected to UUO for 7 days. The experiment was repeated twice. *H*, NAD^+^ levels in kidneys from wildtype or *Cd38*^−/−^ mice subjected to UUO for 7 days (n = 6). The experiment was repeated three times. All data represent the mean ± SEM. ∗∗*p* < 0.01, ∗∗∗*p* < 0.001. *p* values were calculated by two-tailed Student’s *t* test (*D*, *E* and *H*). UUO, unilateral ureteral obstruction.

Given that CD38 was the major NADase that was significantly upregulated in both patients and UUO mice (Fig. 3, *D* and *E*) and CD38 was positively correlated with fibrosis markers (Fig. 3, *F* and *G*), we sought to determine the expression patterns of CD38 and whether CD38 contributed to NAD^+^ decline in obstructive nephropathy. In line with proteomics, Western blots confirmed the elevated levels of CD38 in human and mouse obstructed kidneys (Fig. 4, *A* and *B*). Healthy kidneys barely expressed CD38, whereas immunohistochemistry analysis of kidneys from patients with obstructive nephropathy showed accumulated CD38-expressing cells, which were mainly located in the renal interstitium but not in tubular cells or glomeruli (Fig. 4*C*). Moreover, CD38-positive cells were dramatically increased in UUO kidneys as well (Fig. 4*D*). To further characterize the expression patterns of CD38, we utilized a single cell RNA sequencing (scRNA-seq) dataset of mouse UUO kidneys (GSE140023) (33). We learned that CD38 was mainly expressed in endothelial cells, macrophages, and dendritic cells during renal fibrosis (supplemental Fig. S5, *A*–*C*). Flow cytometry analysis verified the accumulation of CD38-positive immune cells in UUO kidneys (Fig. 4*E*), among which 53.1% were macrophages (Fig. 4*F*). In addition, we also observed CD38 expression in lymphocytes in mouse obstructed kidneys (Fig. 4*F*). Taken together, these data indicate that CD38 is elevated and is partially expressed in immune cells during obstructive nephropathy.

CD38 deficiency increases renal NAD^+^ content in mice under normal conditions (34). We next determined the impact of CD38 on renal NAD^+^ levels in obstructive nephropathy by using *Cd38*^−/−^ mice (35). The absence of CD38 at the protein level in obstructed kidneys was confirmed by flow cytometry (Fig. 4*G*). Importantly, the obstructed kidneys from *Cd38*^−/−^ mice showed a significant rise in NAD^+^ levels compared with wildtype mice (Fig. 4*H*), demonstrating that the fibrosis-associated CD38 upregulation contributed to the decline of renal NAD^+^ levels during obstructive nephropathy.

### CD38 Deletion or Inhibition Ameliorated UUO-Induced Renal Fibrosis

**Fig. 5:**
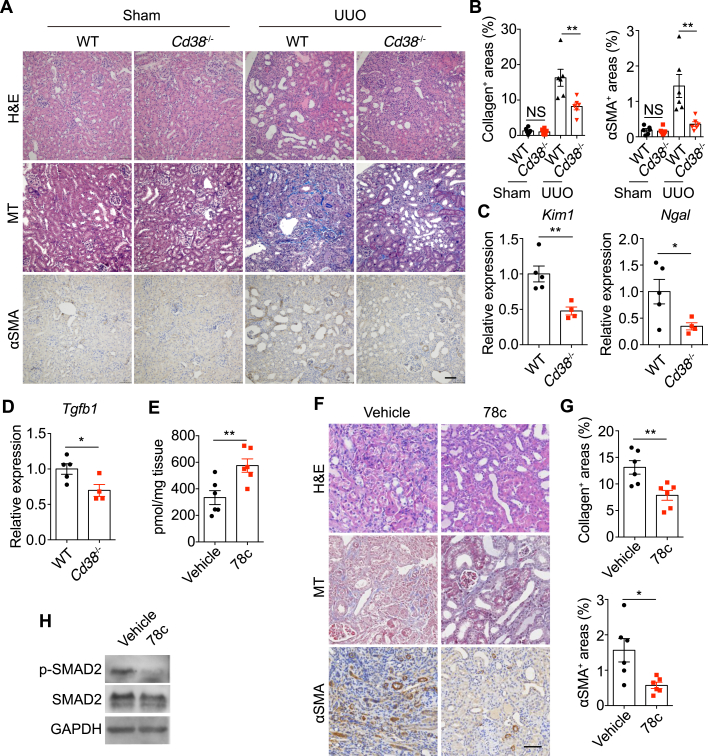
**CD38 deletion or inhibition ameliorated UUO-induced renal fibrosis.***A*, representative images of H&E staining (*upper panels*), MT staining (*middle panels*), and αSMA immunohistochemistry staining (*lower panels*) of kidneys from wildtype and *Cd38*^−/−^ mice subjected to sham or UUO for 7 days (n = 6). The experiment was repeated three times. The scale bar represents 50 μm. *B*, quantification of collagen-positive areas and αSMA-positive areas in (*A*) (n = 6). *C* and *D*, mRNA levels of *Kim1*, *Ngal* (*C*), and *Tgfb1* (*D*) of obstructed kidneys from wildtype and *Cd38*^−/−^ mice subjected to UUO for 7 days (n = 5/4). *E*, NAD^+^ levels in obstructed kidneys from vehicle- or 78c-treated wildtype mice subjected to UUO for 7 days (n = 6). *F*, representative images of H&E staining (*upper panels*), MT staining (*middle panels*), and αSMA immunohistochemistry staining (*lower panels*) of kidneys from vehicle- or 78c-treated wildtype mice subjected to UUO for 7 days (n = 6). The experiment was repeated three times. The scale bar represents 50 μm. *G*, quantification of collagen-positive areas and αSMA-positive areas in (*F*) (n = 6). *H*, immunoblot analysis of phosphorylated (p-) and total protein of SMAD2 in kidney samples from vehicle- and 78c-treated mice subjected to UUO for 7 days. The experiment was repeated three times. All data represent the mean ± SEM. ∗*p* < 0.05, ∗∗*p* < 0.01. *p* values were calculated by two-tailed Student’s *t* test (*B*–*E* and *G*). MT, Masson's trichrome; UUO, unilateral ureteral obstruction.

Since elevated CD38 was positively correlated with fibrosis markers (Fig. 3, *F* and *G*) and contributed to declined renal NAD^+^ levels (Fig. 4*H*), we then attempted to investigate the pathogenic role of CD38 in obstructive nephropathy. Histological examination showed that genetic deletion of CD38 ameliorated UUO-induced renal fibrosis, as evidenced by the decrease in collagen accumulation and αSMA-positive cells (Fig. 5, *A* and *B*). The kidney injury markers, *Kim1* and *Ngal*, were dramatically downregulated in *Cd38*^−/−^ mice, suggesting a protective role of CD38 deletion against obstruction-induced tubular damage (Fig. 5*C*). Moreover, compared with wildtype mice, *Tgfb1* had a ∼30% decrease in UUO kidneys from *Cd38*^−/−^ mice (Fig. 5*D*). Collectively, these results suggest that CD38 promotes UUO-induced renal fibrosis.

Next, we explored the therapeutic potential of targeting CD38 in obstructive nephropathy. 78c is a specific and potent CD38 inhibitor, which is able to induce tissue NAD^+^ boosting through the inhibition of the catalytic activity of CD38 (36). We found that 78c significantly restored NAD^+^ levels in UUO kidneys (Fig. 5*E*). Importantly, 78c treatment diminished collagen deposition and αSMA expression in obstructed kidneys (Fig. 5, *F* and *G*). Moreover, 78c treatment blunted TGF-β signaling by suppressing SMAD2 phosphorylation in UUO kidneys (Fig. 5*H*). These data suggest that CD38 inhibition mitigates obstruction-induced renal fibrosis.

### NAD^+^ Supplementation Blunted UUO-Induced Renal Fibrosis

**Fig. 6:**
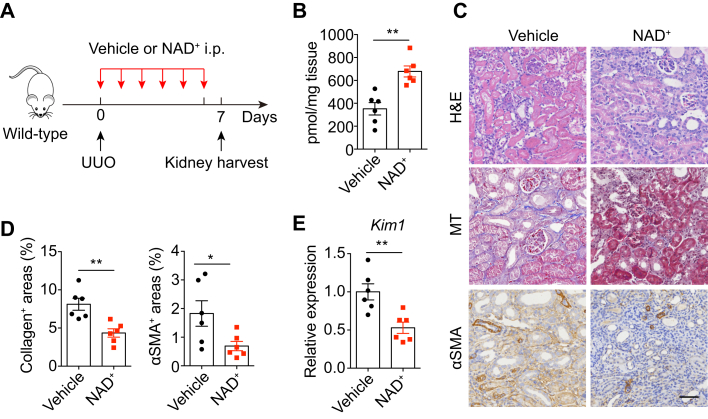
**NAD**^**+**^**supplementation blunted UUO-induced renal fibrosis.***A*, schematic of the experimental design. Eight-week-old male wildtype C57BL/6 mice were subjected to UUO operation and were injected with vehicle or NAD^+^ peritoneally once a day before kidney harvest at day 7 post UUO. *B*, NAD^+^ levels in kidneys from vehicle- and NAD^+^-treated mice subjected to UUO for 7 days (n = 6). *C*, representative images of obstructed kidneys from vehicle- and NAD^+^-treated UUO mice were stained for H&E, Masson's trichrome, and αSMA (n = 6). The experiment was repeated three times. The scale bar represents 50 μm. *D*, quantitation of the collagen-positive areas and αSMA-positive areas of kidney sections in (*C*). *E*, mRNA levels of *Kim1* in obstructed kidneys from vehicle- or NAD^+^-treated UUO mice (n = 6). All data represent the mean ± SEM. ∗*p* < 0.05, ∗∗*p* < 0.01. *p* values were calculated by two-tailed Student’s *t* test (*B*, *D* and *E*). UUO, unilateral ureteral obstruction.

Having uncovered the NAD^+^-degrading and profibrotic role of CD38 in obstructive nephropathy, we then asked whether NAD^+^ supplementation can confer protective effects against obstruction-induced renal fibrosis. We injected NAD^+^ peritoneally for six consecutive days into wildtype mice subjected to UUO operation, and kidneys were harvested 7 days later (Fig. 6*A*). NAD^+^ administration significantly increased tissue levels of NAD^+^ in obstructed kidneys (Fig. 6*B*). Histologically, collagen accumulation and αSMA expression were attenuated in UUO kidneys from NAD^+^-treated mice, whereas their levels were much higher in vehicle-treated mice (Fig. 6, *C* and *D*). Moreover, NAD^+^ supplementation downregulated the expression of kidney injury marker *Kim1* in UUO kidneys (Fig. 6*E*). These data indicate that NAD^+^ protects kidneys from UUO-induced renal fibrosis.

### Deletion or Inhibition of CD38 and NAD^+^ Supplementation Reduced Inflammation in Obstructed Kidneys

**Fig. 7:**
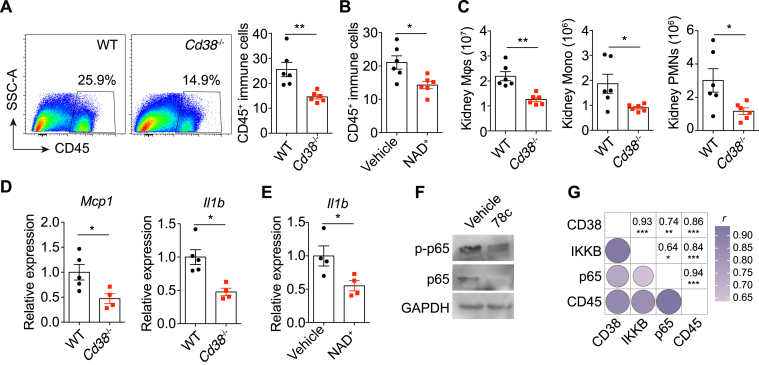
**Deletion or inhibition of CD38 and NAD**^**+**^**supplementation reduced inflammation in obstructed kidneys.***A*, flow cytometry analysis and quantification showing the infiltration of CD45^+^ cells in obstructed kidneys from wildtype and *Cd38*^−/−^ mice subjected to UUO for 7 days (n = 6). *B*, quantification of the infiltration of CD45^+^ cells in obstructed kidneys from UUO mice received vehicle or NAD^+^ treatment (n = 6). *C*, numbers of macrophages (Mφs), monocytes (Mono), and neutrophils (PMNs) in obstructed kidneys from wildtype and *Cd38*^−/−^ mice subjected to UUO for 7 days (n = 6). *D*, mRNA levels of *Mcp1* and *Il1b* in obstructed kidneys from wildtype and *Cd38*^−/−^ mice subjected to UUO for 7 days (n = 5/4). *E*, mRNA levels of *Il1b* in obstructed kidneys from UUO mice that received vehicle or NAD^+^ treatment (n = 4). *F*, immunoblot analysis of phosphorylated (p-) and total protein of NF-κB p65 in kidney samples from vehicle- and 78c-treated mice subjected to UUO for 7 days. Representative data of three separate experiments. *G*, Spearman's rank correlation between the expression of IKKB, NF-κB p65, CD45, and CD38 in UUO kidneys. The data were extracted from mouse kidney proteomes. All data represent the mean ± SEM. ∗*p* < 0.05, ∗∗*p* < 0.01, ∗∗∗*p* < 0.001. *p* values were calculated by two-tailed Student’s *t* test (*A*–*E*) and Spearman's rank correlation analysis (*G*). UUO, unilateral ureteral obstruction.

Next, we asked the mechanisms by which CD38 and NAD^+^ decline promoted renal fibrosis induced by obstruction. Since CD38 and NAD^+^ are known for their roles in regulating immune response (17) and considerable evidence demonstrates that unresolved inflammation plays a substantial role in kidney fibrosis development (7, 25), we then determined the impact of CD38 and NAD^+^ on kidney inflammation. We found that CD38 deletion and NAD^+^ supplementation significantly decreased the recruitment of immune cells into UUO kidneys (Fig. 7, *A* and *B*). The obstructed kidneys from *Cd38*^−/−^ mice recruited fewer macrophages, monocytes, and neutrophils compared with wildtype mice (Fig. 7*C*). Interestingly, the obstructed kidneys from *Cd38*^−/−^ mice expressed a lower level of *Mcp1* (Fig. 7*D*), encoding monocyte chemotactic protein 1, which is essential to recruit monocytes/macrophages into inflamed tissue (37). Moreover, scRNA-seq analysis revealed significant enrichment of leukocyte migration and cell adhesion in CD38-positive macrophages (supplemental Fig. S5*D*), suggesting that CD38 was required for the recruitment of immune cells. Besides, the proinflammatory cytokine IL-1β was significantly downregulated in obstructed kidneys after CD38 deletion (Fig. 7*D*) and NAD^+^ supplementation (Fig. 7*E*). CD38 and NAD^+^ decline are also associated with activated NF-κB signaling, which is involved in the development of renal fibrosis (25, 38). Notably, CD38 inhibition blunted NF-κB signaling by downregulating the expression of NF-κB p65 and its phosphorylation (Fig. 7*F*). In line with these results, the mouse kidney proteomics showed that the expression of CD38 was positively correlated with the abundance of CD45 (*r* = 0.86, *p* < 0.001), IKKB (*r* = 0.93, *p* < 0.001), and NF-κB p65 (*r* = 0.74, *p* < 0.01) (Fig. 7*G*). Taken together, these results indicate that targeting CD38 and NAD^+^ metabolism reduces kidney inflammation, partially by suppressing the infiltration of immune cells and NF-κB signaling, thus mitigating obstruction-induced renal fibrosis.

## Discussion

Considerable advances in understanding obstruction-induced renal fibrosis have been made, yet most of them were based on animal experiments and a comprehensive understanding of human obstructive nephropathy at the proteome level is lacking. In the current study, we presented a proteomic landscape of human obstructed kidneys allowing the analysis and extraction of altered signaling pathways at the proteome level. Importantly, the proteomics uncovered the previously underexplored dysregulation of NAD^+^ metabolism in obstructed kidneys. Moreover, the major NAD consumer CD38 was strongly induced in human and experimental obstructive nephropathy, which led to declined renal NAD^+^ levels. CD38 deletion or inhibition and NAD^+^ supplementation restored NAD^+^ levels and ameliorated UUO-induced renal fibrosis, partially through the mechanism of reducing kidney inflammation. Our study provides solid molecular evidence at the proteome level for the first time derived directly from pediatric patients with obstructive nephropathy.

Several studies implied that NAD^+^ levels are associated with diabetic nephropathy (10, 39, 40), whereas little is known about their roles in obstructive nephropathy. Our study directly addressed the aberrant NAD^+^ metabolism in human obstructed kidneys at the proteome level. Moreover, NAD^+^ restoration by NAD^+^ supplementation protects renal fibrosis in UUO mice, which was consistent with a previous study showing that supplementation of the NAD^+^ precursor NAM inhibited UUO-induced renal fibrosis, although NAD^+^ levels were not assessed after NAM treatment in this study (41). Therefore, NAD^+^ boosting might represent an optional therapy for obstructive nephropathy. Several clinical trials are currently ongoing to test the therapeutic potential of NAD^+^ boosters in human kidney diseases, some of which showed clinical benefits, while others did not (10), indicating that challenges remain for their clinical translation. Supplementation of NAD^+^ precursors and inhibition of NAD^+^ consumers are common strategies to recover NAD^+^ levels. In the present study, the observation of defective NAD^+^ synthesis in obstructed kidneys raised the possibility that precursor supplementation could be less effective. Importantly, the major NADase CD38 was strongly induced in human and experimental obstructive nephropathy, and its deletion or inhibition recovered renal NAD^+^ levels and ameliorated UUO-induced renal fibrosis. This was consistent with a recent study demonstrating that targeting CD38-dependent NAD^+^ degradation mitigates skin, lung, and peritoneal fibrosis (42). These findings suggest that restoration of NAD^+^ levels by inhibiting NAD^+^ consumers (for example, CD38) might be a therapeutic strategy for obstructive nephropathy.

CD38 is expressed by various cell types and can be induced during inflammatory conditions, especially in hematopoietic cells (17, 43). We found that CD38 was partially expressed in immune cells during UUO-induced renal fibrosis, half of which were CD11b^+^ F4/80^+^ macrophages, suggesting that a subset of CD38-positive macrophages emerged during the fibrotic process. A study reported lately that CD38 on macrophages is capable of reducing tissue levels of NAD^+^ during aging, depending on its ectoenzyme activity (18). Moreover, CD38 can modulate inflammation by regulating cell recruitment, phagocytosis, and cytokine release (17). Our work showed that CD38 was required for the infiltration of innate immune cells and IL-1β expression. Of note, emerging evidence supports the idea that CD38-mediated NAD^+^ depletion contributes to the inflammatory response (17). In our hand, inhibition of CD38 catalytic activity by 78c blunted NF-κB signaling, suggesting that the immunomodulatory role of CD38 was associated with its NAD^+^-degrading activity. Besides, we cannot exclude the contribution of CD38 expressed in other cells (for example, endothelial cells) to NAD^+^ consumption and kidney pathologies.

The kidney is a high energy-demanding organ that maintains the homeostasis of electrolyte, water, and acid–base balance, requiring a large number of mitochondria to meet energy needs. A correlation between mitochondrial dysfunction and kidney diseases has been demonstrated repeatedly (44). Our kidney proteomics revealed a global dysregulation of cellular metabolism in obstructed kidneys, and mitochondrial abnormalities were central to these metabolic alterations. Moreover, CD38 increase in aging mice contributes to the development of mitochondrial dysfunction, by degrading cellular NAD^+^ and subsequently suppressing SIRT3 (45). SIRT3 is one of the sirtuins localized in mitochondria that regulate key mitochondrial proteins important for oxidative homeostasis (46). In our study, we observed a dramatic downregulation of SIRT3 in obstructed kidneys. Therefore, mitochondrial abnormalities in obstructive nephropathy might be attributed to CD38 elevation, NAD^+^ decline, and SIRT3 suppression. Targeting CD38 and NAD^+^ metabolism might restore mitochondrial function, which needs to be further investigated.

In summary, we presented a proteomic landscape of obstructed kidneys from pediatric patients with UPJO and provided a rich resource of proteomic data to facilitate future study of obstructive nephropathy. We uncovered an aberrant NAD^+^ metabolism and a fibrosis-associated elevation of CD38 during obstructive nephropathy. CD38 deletion or inhibition and NAD^+^ supplementation mitigated UUO-induced renal fibrosis, partially through the mechanism of reducing kidney inflammation. Thus, our study emphasized the importance and therapeutic potential of CD38-mediated NAD^+^ metabolism in obstructive nephropathy.

### Limitations of This Study

A limitation of the present study is the small size of the patient samples, for the reason that it is difficult to recruit a large number of pediatric patients. Besides, there are not technical or biological replicates for the DIA-based proteomics analysis. However, this can be partially compensated by a further in-depth exploration of the main findings. Since CD38 is barely expressed in tubular cells, further investigations are required to identify how CD38-expressing immune cells affect NAD^+^ levels and metabolic events in tubular cells. Moreover, the mechanisms by which CD38 and CD38-mediated NAD^+^ decline regulate kidney inflammation and fibrosis remain to be elucidated.

## Data Availability

The MS raw data have been deposited to the ProteomeXchange Consortium *via* the iProx partner repository (47, 48) with the dataset identifier PXD039314.

## Supplemental data

This article contains supplemental data.

## Conflict of interest

The authors declare no competing interests.

## Supplemental Data

Supplemental Data 3

Supplemental Fig. S1 **Proteomic landscape of human obstructed kidneys.***A*–*C*, heatmap indicating the expression of identified proteins related to collagens (*A*), phagocytosis, cytokine signaling in immune response (*B*), and interferon signaling (*C*) in human control and obstructed kidneys. *D*, immunoblot analysis of phosphorylated (p-) and total protein of SMAD2 in kidney samples from controls and patients with obstructive nephropathy. Represented results of three separate experiments. **Supplemental Fig. S2**. **Mitochondrial dysfunction in human obstructed kidneys.** Heatmap indicating the expression of significant down-regulated proteins (*p* value <0.05, *q* value <0.05, and Ob/Ctr fold change <0.5) related to mitochondria organization in human obstructed kidneys. **Supplemental Fig. S3**. **Proteomic profiling of mouse obstructed kidneys.***A*, PCA analysis of sham kidney proteomes and UUO kidney proteomes (n = 6/6). *B*, heatmap showing all proteins identified by DIA-MS of kidneys from wild-type mice subjected to sham or UUO operation for 7 days. *C* and *D*, top 20 most significant terms enriched by significantly up-regulated (*C*) and down-regulated (*D*) proteins (*p* value <0.05, *q* value <0.05, and UUO/Sham fold change >2 or <0.5) in obstructed kidneys from UUO mice. **Supplemental Fig. S4**. **Aberrant NAD**^**+**^**metabolism in human and mouse obstructed kidneys.***A* and *B*, expression levels of key enzymes catalyzing NAD^+^ synthesis and consumption in human (*A*) and mouse (*B*) kidney proteomics. All data represent the mean ± SEM. ∗*p* < 0.05, ∗∗*p* < 0.01, ∗∗∗*p* < 0.01, NS no significance. *p* values were calculated by two-tailed Student’s *t*-test. **Supplemental Fig. S5**. **The expression patterns of CD38 in UUO kidneys.***A*, Uniform Manifold Approximation and Projection (UMAP) dimension reduction showing distinct cell types identified by unsupervised clustering. The scRNAseq data were from Gene Expression Omnibus (GEO) database (GSE140023). PCT, proximal convoluted tubule; Mac, macrophage; Neu, neutrophil; Mono, monocyte; DC, dendritic cell; EC, endothelial cell; NK, natural killer cell; Mes, mesenchymal cell; LoH, loop of Henle; CD, Collecting Duct. *B* and *C*, feature plots (*B*) and violin plots (*C*) showing the expression of *Cd38* in each cell type in UUO kidneys. *D*, GO analysis showing the top 20 upregulated pathways in CD38^+^ macrophages. **Supplemental Fig. S6**. **Uncropped scans of Western blots**.
