# Supplementary material for: Comprehensive Proteomics Analysis Identifies CD38-Mediated NAD+ Decline Orchestrating Renal Fibrosis in Pediatric Patients With Obstructive Nephropathy
Source: Mol Cell Proteomics. 2023 Feb 17;22(3):100510. doi: 10.1016/j.mcpro.2023.100510 (PMC10025283; doi:10.1016/j.mcpro.2023.100510)

Figure S1

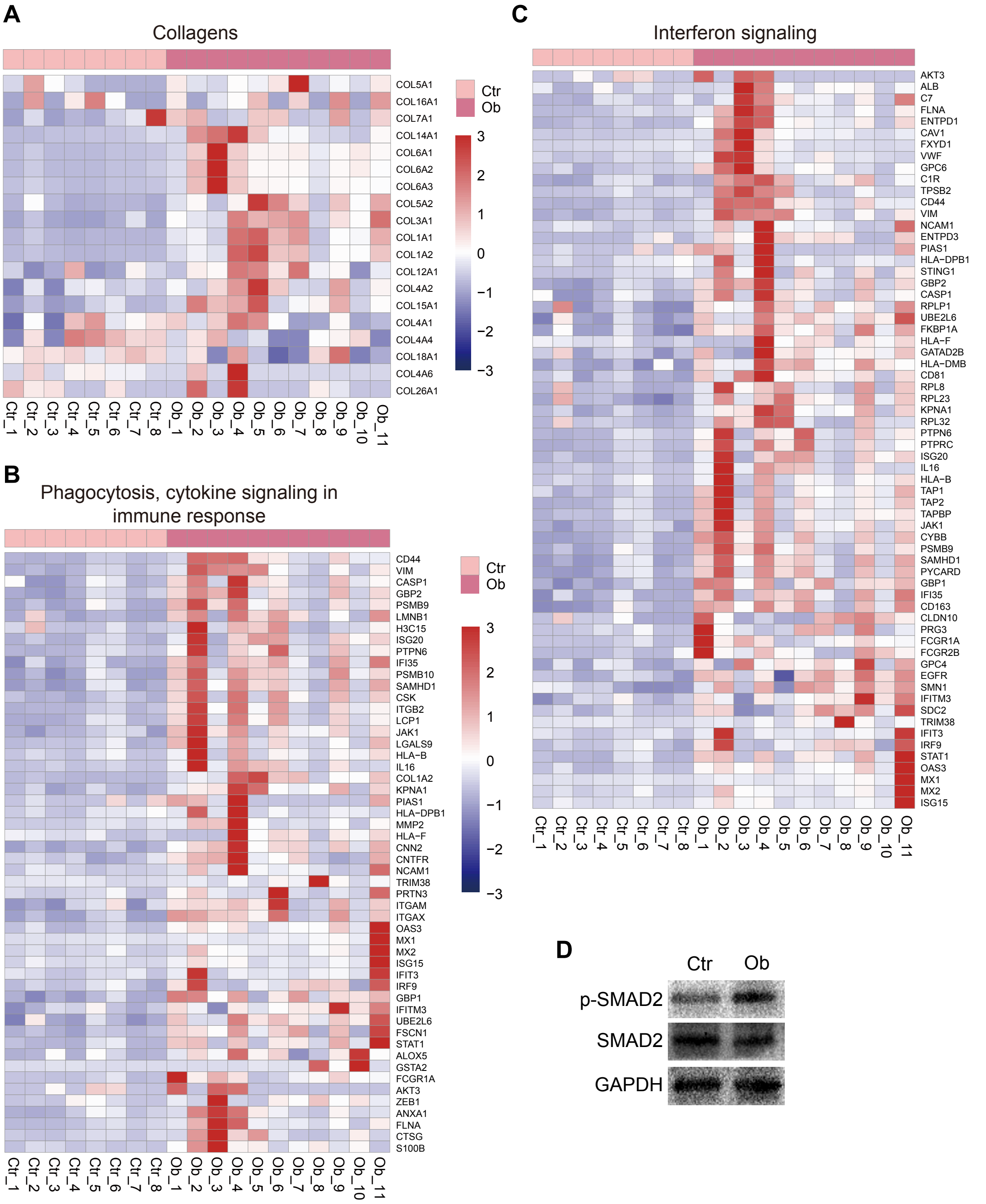
A
Collagens
Ctr
Ob
3
2
1
0
−1
−2
−3
COL5A1
COL16A1
COL7A1
COL14A1
COL6A1
COL6A2
COL6A3
COL5A2
COL3A1
COL1A1
COL1A2
COL12A1
COL4A2
COL15A1
COL4A1
COL4A4
COL18A1
COL4A6
COL26A1
Ctr_1
Ctr_2
Ctr_3
Ctr_4
Ctr_5
Ctr_6
Ctr_7
Ctr_8
Ob_1
Ob_2
Ob_3
Ob_4
Ob_5
Ob_6
Ob_7
Ob_8
Ob_9
Ob_10
Ob_11
B
Phagocytosis, cytokine signaling in immune response
Ctr
Ob
3
2
1
0
−1
−2
−3
CD44
VIM
CASP1
GBP2
PSMB9
LMNB1
H3C15
ISG20
PTPN6
IFI35
PSMB10
SAMHD1
CSK
ITGB2
LCP1
JAK1
LGALS9
HLA−B
IL16
COL1A2
KPNA1
PIAS1
HLA−DPB1
MMP2
HLA−F
CNN2
CNTFR
NCAM1
TRIM38
PRTN3
ITGAM
ITGAX
OAS3
MX1
MX2
ISG15
IFIT3
IRF9
GBP1
IFITM3
UBE2L6
FSCN1
STAT1
ALOX5
GSTA2
FCGR1A
AKT3
ZEB1
ANXA1
FLNA
CTSG
S100B
Ctr_1
Ctr_2
Ctr_3
Ctr_4
Ctr_5
Ctr_6
Ctr_7
Ctr_8
Ob_1
Ob_2
Ob_3
Ob_4
Ob_5
Ob_6
Ob_7
Ob_8
Ob_9
Ob_10
Ob_11
C
Interferon signaling
Ctr
Ob
3
2
1
0
−1
−2
−3
AKT3
ALB
C7
FLNA
ENTPD1
CAV1
FXYD1
VWF
GPC6
C1R
TPSB2
CD44
VIM
NCAM1
ENTPD3
PIAS1
HLA−DPB1
STING1
GBP2
CASP1
RPLP1
UBE2L6
FKBP1A
HLA−F
GATAD2B
HLA−DMB
CD81
RPL8
RPL23
KPNA1
RPL32
PTPN6
PTPRC
ISG20
IL16
HLA−B
TAP1
TAP2
TAPBP
JAK1
CYBB
PSMB9
SAMHD1
PYCARD
GBP1
IFI35
CD163
CLDN10
PRG3
FCGR1A
FCGR2B
GPC4
EGFR
SMN1
IFITM3
SDC2
TRIM38
IFIT3
IRF9
STAT1
OAS3
MX1
MX2
ISG15
Ctr_1
Ctr_2
Ctr_3
Ctr_4
Ctr_5
Ctr_6
Ctr_7
Ctr_8
Ob_1
Ob_2
Ob_3
Ob_4
Ob_5
Ob_6
Ob_7
Ob_8
Ob_9
Ob_10
Ob_11
D
Ctr
Ob
p-SMAD2
SMAD2
GAPDH

Figure S2

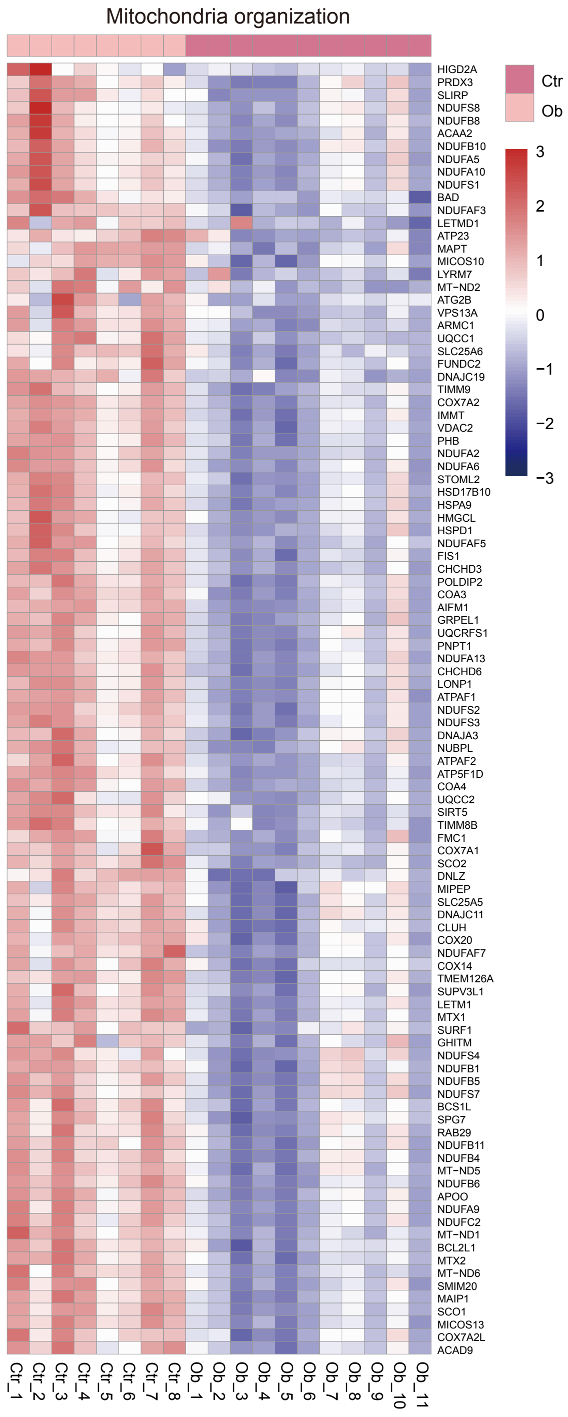
Mitochondria organization
HIGD2A
PRDX3
SLIRP
NDUFS8
NDUFB8
ACAA2
NDUFB10
NDUFA5
NDUFA10
NDUFS1
BAD
NDUFAF3
LETMD1
ATP23
MAPT
MICOS10
LYRM7
MT−ND2
ATG2B
VPS13A
ARMC1
UQCC1
SLC25A6
FUNDC2
DNAJC19
TIMM9
COX7A2
IMMT
VDAC2
PHB
NDUFA2
NDUFA6
STOML2
HSD17B10
HSPA9
HMGCL
HSPD1
NDUFAF5
FIS1
CHCHD3
POLDIP2
COA3
AIFM1
GRPEL1
UQCRFS1
PNPT1
NDUFA13
CHCHD6
LONP1
ATPAF1
NDUFS2
NDUFS3
DNAJA3
NUBPL
ATPAF2
ATP5F1D
COA4
UQCC2
SIRT5
TIMM8B
FMC1
COX7A1
SCO2
DNLZ
MIPEP
SLC25A5
DNAJC11
CLUH
COX20
NDUFAF7
COX14
TMEM126A
SUPV3L1
LETM1
MTX1
SURF1
GHITM
NDUFS4
NDUFB1
NDUFB5
NDUFS7
BCS1L
SPG7
RAB29
NDUFB11
NDUFB4
MT−ND5
NDUFB6
APOO
NDUFA9
NDUFC2
MT−ND1
BCL2L1
MTX2
MT−ND6
SMIM20
MAIP1
SCO1
MICOS13
COX7A2L
ACAD9
Ctr_1
Ctr_2
Ctr_3
Ctr_4
Ctr_5
Ctr_6
Ctr_7
Ctr_8
Ob_1
Ob_2
Ob_3
Ob_4
Ob_5
Ob_6
Ob_7
Ob_8
Ob_9
Ob_10
Ob_11
Ctr
Ob
3
2
1
0
−1
−2
−3

Figure S3

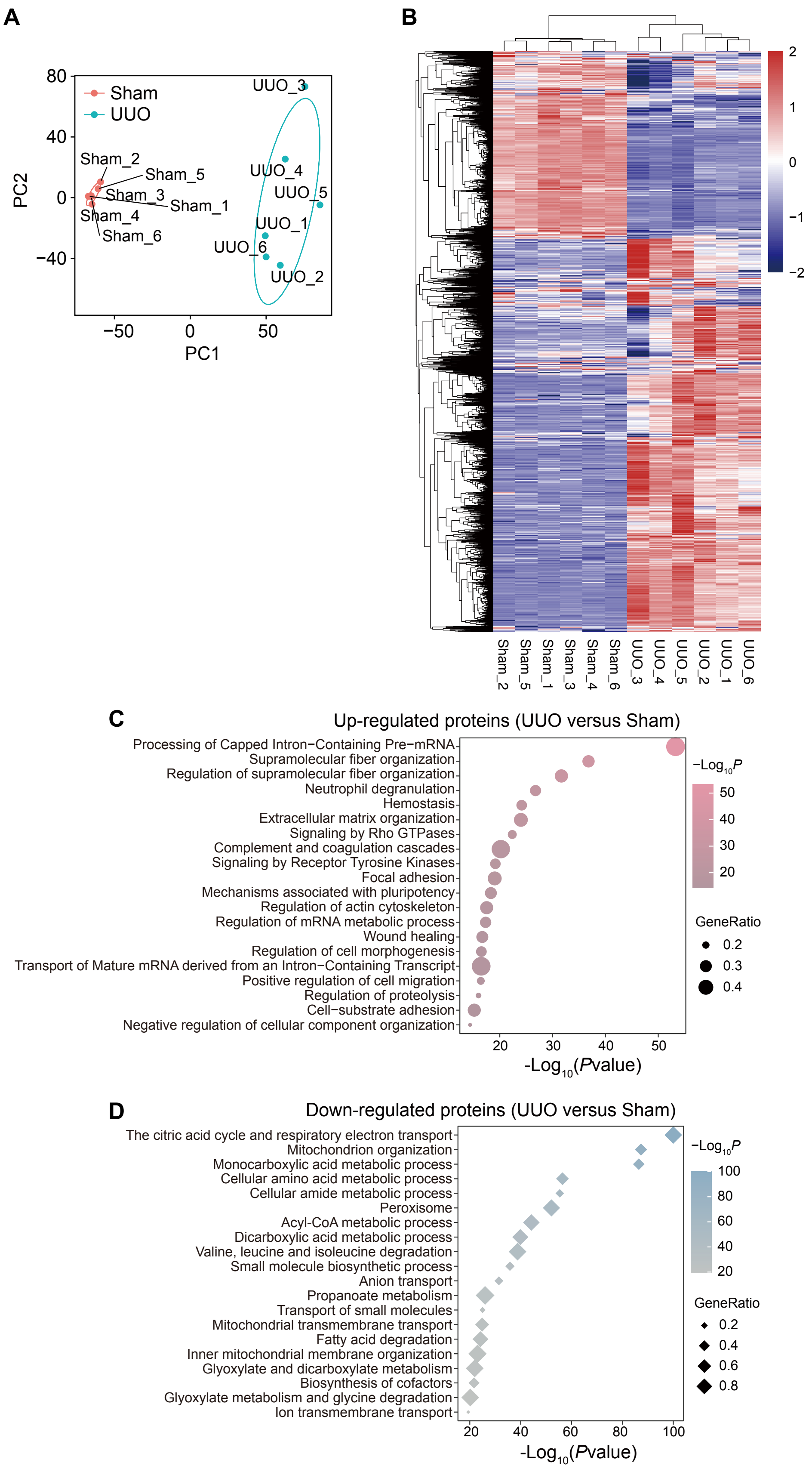
A
Sham
UUO
PC1
PC2
Sham_2
Sham_5
Sham_3
Sham_1
Sham_4
Sham_6
UUO_3
UUO_4
UUO_5
UUO_1
UUO_6
UUO_2
B
Sham_2
Sham_5
Sham_1
Sham_3
Sham_4
Sham_6
UUO_3
UUO_4
UUO_5
UUO_2
UUO_1
UUO_6
C
Up-regulated proteins (UUO versus Sham)
Processing of Capped Intron−Containing Pre−mRNA
Supramolecular fiber organization
Regulation of supramolecular fiber organization
Neutrophil degranulation
Hemostasis
Extracellular matrix organization
Signaling by Rho GTPases
Complement and coagulation cascades
Signaling by Receptor Tyrosine Kinases
Focal adhesion
Mechanisms associated with pluripotency
Regulation of actin cytoskeleton
Regulation of mRNA metabolic process
Wound healing
Regulation of cell morphogenesis
Transport of Mature mRNA derived from an Intron−Containing Transcript
Positive regulation of cell migration
Regulation of proteolysis
Cell−substrate adhesion
Negative regulation of cellular component organization
−Log10P
GeneRatio
-Log10(Pvalue)
D
Down-regulated proteins (UUO versus Sham)
The citric acid cycle and respiratory electron transport
Mitochondrion organization
Monocarboxylic acid metabolic process
Cellular amino acid metabolic process
Cellular amide metabolic process
Peroxisome
Acyl-CoA metabolic process
Dicarboxylic acid metabolic process
Valine, leucine and isoleucine degradation
Small molecule biosynthetic process
Anion transport
Propanoate metabolism
Transport of small molecules
Mitochondrial transmembrane transport
Fatty acid degradation
Inner mitochondrial membrane organization
Glyoxylate and dicarboxylate metabolism
Biosynthesis of cofactors
Glyoxylate metabolism and glycine degradation
Ion transmembrane transport
−Log10P
GeneRatio
-Log10(Pvalue)

Figure S4

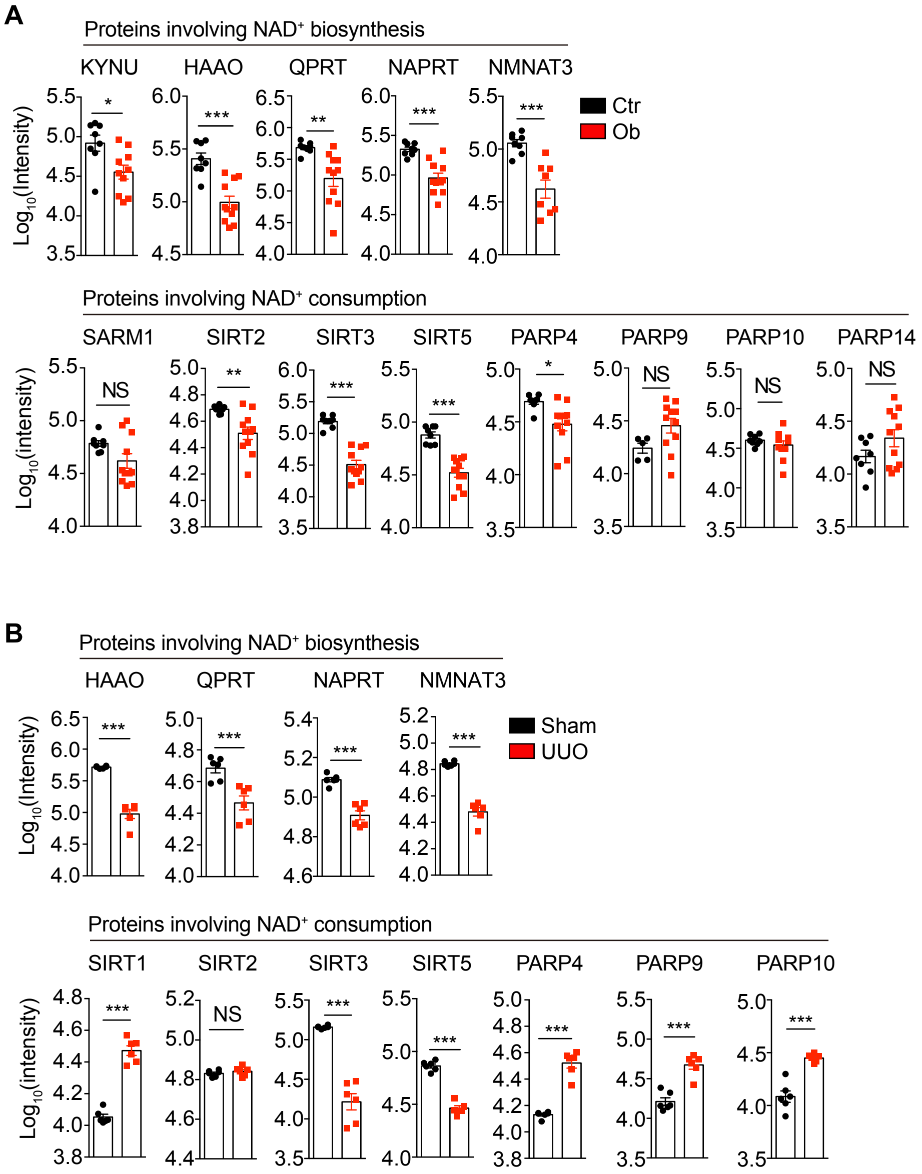
A
Proteins involving NAD+ biosynthesis
KYNU
HAAO
QPRT
NAPRT
NMNAT3
Ctr
Ob
Log10(Intensity)
Proteins involving NAD+ consumption
SARM1
SIRT2
SIRT3
SIRT5
PARP4
PARP9
PARP10
PARP14
Log10(intensity)
NS
B
Proteins involving NAD+ biosynthesis
HAAO
QPRT
NAPRT
NMNAT3
Sham
UUO
Log10(Intensity)
Proteins involving NAD+ consumption
SIRT1
SIRT2
SIRT3
SIRT5
PARP4
PARP9
PARP10
Log10(intensity)
NS

Figure S5

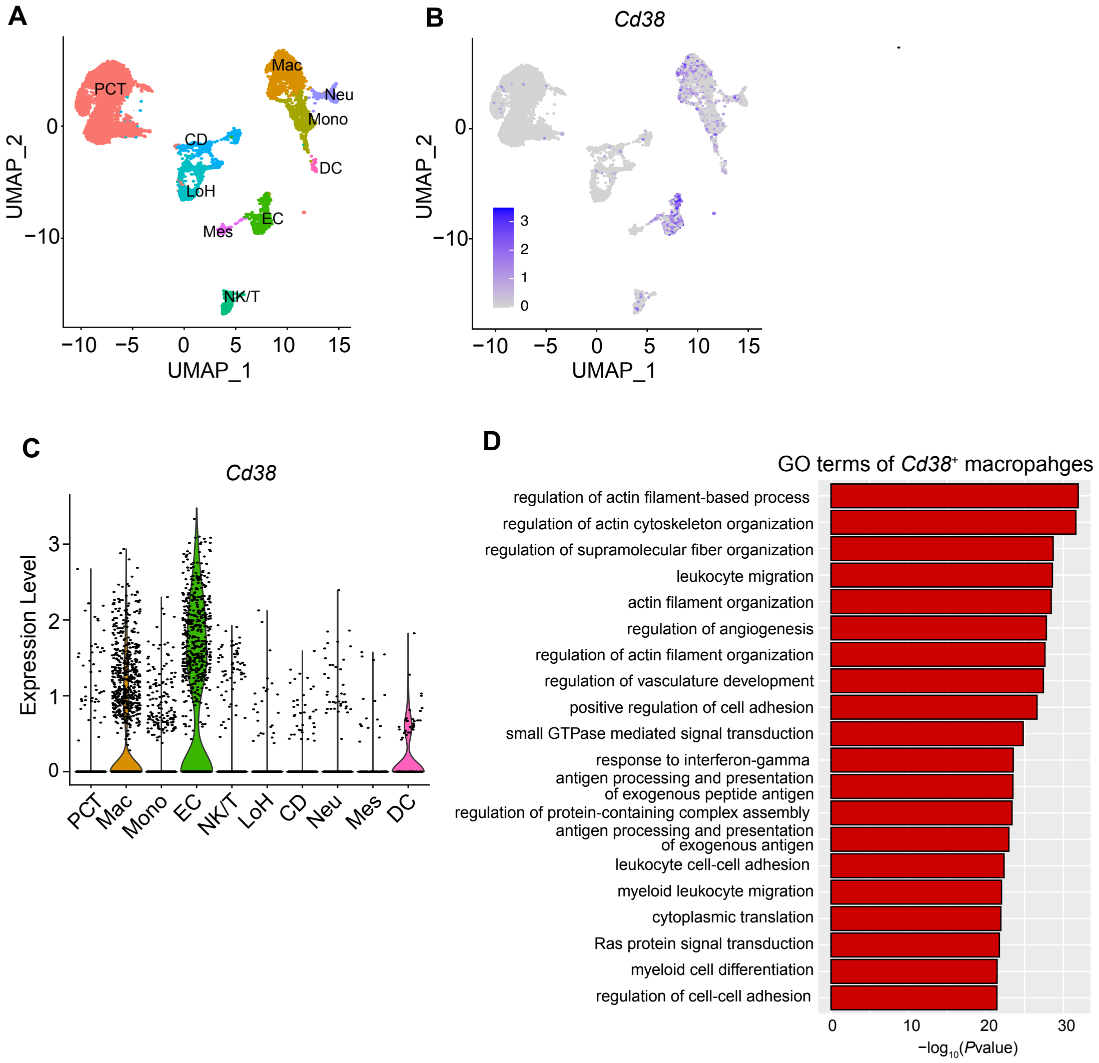
A
PCT
CD
LoH
Mac
Neu
Mono
DC
EC
Mes
NK/T
UMAP_2
UMAP_1
B
Cd38
C
Cd38
Expression Level
PCT
Mac
Mono
EC
NK/T
LoH
CD
Neu
Mes
DC
D
GO terms of Cd38+ macropahges
regulation of actin filament-based process
regulation of actin cytoskeleton organization
regulation of supramolecular fiber organization
leukocyte migration
actin filament organization
regulation of angiogenesis
regulation of actin filament organization
regulation of vasculature development
positive regulation of cell adhesion
small GTPase mediated signal transduction
response to interferon-gamma
antigen processing and presentation of exogenous peptide antigen
regulation of protein-containing complex assembly
antigen processing and presentation of exogenous antigen
leukocyte cell-cell adhesion
myeloid leukocyte migration
cytoplasmic translation
Ras protein signal transduction
myeloid cell differentiation
regulation of cell-cell adhesion
−log10(Pvalue)

Figure S6 Uncropped scans of western blots

Related to Figure 4A

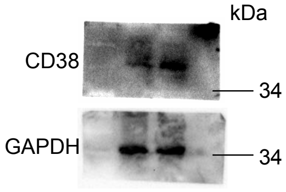

Related to Figure 4B

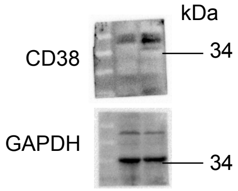

Related to Figure 5H

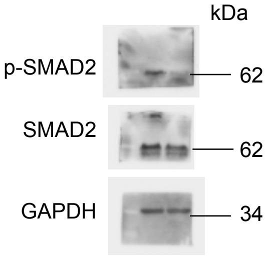

Related to Figure 7F

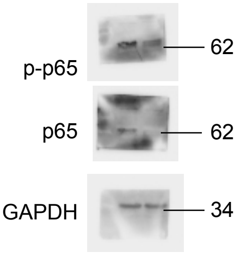

Related to Figure S1D

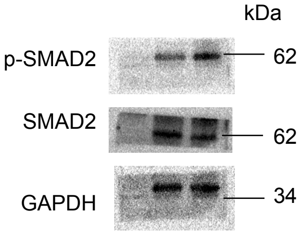

Supplement: Supplemental Fig. S1 — Proteomic landscape of human obstructed kidneys.A–C, heatmap indicating the expression of identified proteins related to collagens (A), phagocytosis, cytokine signaling in immune response (B), and interferon signaling (C) in human control and obstructed kidneys. D, immunoblot analysis of phosphorylated (p-) and total protein of SMAD2 in kidney samples from controls and patients with obstructive nephropathy. Represented results of three separate experiments. Supplemental Fig. S2. Mitochondrial dysfunction in human obstructed kidneys. Heatmap indicating the expression of significant down-regulated proteins (p value <0.05, q value <0.05, and Ob/Ctr fold change <0.5) related to mitochondria organization in human obstructed kidneys. Supplemental Fig. S3. Proteomic profiling of mouse obstructed kidneys.A, PCA analysis of sham kidney proteomes and UUO kidney proteomes (n = 6/6). B, heatmap showing all proteins identified by DIA-MS of kidneys from wild-type mice subjected to sham or UUO operation for 7 days. C and D, top 20 most significant terms enriched by significantly up-regulated (C) and down-regulated (D) proteins (p value <0.05, q value <0.05, and UUO/Sham fold change >2 or <0.5) in obstructed kidneys from UUO mice. Supplemental Fig. S4. Aberrant NAD+metabolism in human and mouse obstructed kidneys.A and B, expression levels of key enzymes catalyzing NAD+ synthesis and consumption in human (A) and mouse (B) kidney proteomics. All data represent the mean ± SEM. ∗p < 0.05, ∗∗p < 0.01, ∗∗∗p < 0.01, NS no significance. p values were calculated by two-tailed Student’s t-test. Supplemental Fig. S5. The expression patterns of CD38 in UUO kidneys.A, Uniform Manifold Approximation and Projection (UMAP) dimension reduction showing distinct cell types identified by unsupervised clustering. The scRNAseq data were from Gene Expression Omnibus (GEO) database (GSE140023). PCT, proximal convoluted tubule; Mac, macrophage; Neu, neutrophil; Mono, monocyte; DC, dendritic cell; EC, endo [file mmc4.pdf]
